# Music engagement, metacognitions, and performance outcomes: an empirical investigation among Chinese advanced music students

**DOI:** 10.3389/fpsyg.2025.1712501

**Published:** 2025-12-17

**Authors:** Jieru Zhang

**Affiliations:** Henan Conservatoire, Zhengzhou University, Zhengzhou, China

**Keywords:** music engagement, metacognition, performance outcomes, self-regulation, music education

## Abstract

**Background:**

This study investigates the role of engagement and metacognitive factors in predicting performance outcomes among advanced Chinese music students. Drawing on theoretical frameworks in metacognition and self-regulation, the research tests a set of hypotheses examining both direct and mediated pathways linking students’ engagement with performance success. Building on the metacognitive model of Wells and Matthews and the theory of self-regulated learning, this study conceptualizes three dimensions of metacognitive functioning—cognitive confidence, need to control thoughts, and positive beliefs about worry—as cognitive, regulatory, and motivational mediators that explain how engagement translates into performance achievement.

**Methods:**

The study sample consisted of advanced music students from various conservatories in China. Descriptive and correlation analyses were performed using SPSS version 29.0, while structural equation modeling (SEM) was conducted with SmartPLS 4.

**Results:**

Findings support the direct influence of music students’ engagement on performance outcomes, highlighting the importance of proactive involvement in practice. Cognitive confidence is shown to mediate this relationship positively, while the need to control thoughts has a minor mediated effect, and positive beliefs about worry demonstrate a negligible impact.

**Conclusion:**

These findings emphasize the multifaceted role of metacognition in music education, suggesting that metacognitive skills such as planning, monitoring, and self-regulation significantly enhance learning processes and performance. Practical implications include recommendations for music educators to incorporate metacognitive training into their curricula, fostering students’ cognitive awareness and adaptive learning strategies. This study contributes to the growing field of metacognitive research in music education, offering insights into optimizing performance outcomes through targeted engagement and reflective practice.

## 1 Introduction

The complex interplay between engagement in music education and its impact on performance outcomes is a critical area of interest in music psychology and educational research (Burin and Osorio, 2016). For advanced music students, consistent practice and deep involvement in musical activities contribute significantly to their development and proficiency. However, the cognitive processes that underpin such engagement and facilitate the translation of practice into performance success still need to be explored (Cotter et al., 2017), particularly within specific cultural contexts like China. Existing research has highlighted unique aspects of Chinese music education, such as the role of social and educational settings in shaping engagement (Ho and Law, 2006) and the importance of integrating contemporary and traditional musical approaches (Cheung, 2004). This study aims to investigate how metacognitions influence the relationship between music engagement and performance outcomes among advanced Chinese music students. Grounded in the metacognitive model of Wells and Matthews (1994) and the theory of self-regulated learning (Zimmerman and Kitsantas, 1997), the study conceptualizes three metacognitive dimensions—cognitive confidence, need to control thoughts, and positive beliefs about worry—as mechanisms that explain how engagement translates into performance success. Understanding these pathways can inform pedagogical approaches and strategies to optimize students’ musical training and performance.

### 2 Theoretical framework of metacognition in music education

The theoretical foundation of this study is grounded in the metacognitive model proposed by Wells and Matthews (1994), which defines metacognition as the system of beliefs and processes that enable individuals to monitor, control, and regulate their own cognition. This model was further elaborated by Wells and Cartwright-Hatton (2004) and extended to non-clinical settings to explain adaptive and maladaptive self-regulation. In educational contexts, metacognitive regulation allows learners to plan, monitor, and evaluate their actions effectively (Hallam, 2001). Within music education, these processes are essential for mastering complex performance tasks that require simultaneous cognitive, emotional, and motor coordination (Araújo et al., 2024).

The present study also integrates the principles of the theory of self-regulated learning (Zimmerman and Kitsantas, 1997), which situates metacognition at the center of the relationship between motivation and achievement. In this framework, engagement represents the motivational drive that fuels learning efforts, whereas metacognition serves as the cognitive mechanism that converts effort into measurable performance outcomes. Prior research in music education has emphasized that students with higher metacognitive awareness demonstrate superior practice organization, error detection, and expressive interpretation (Concina, 2019; Bonnaire and González-Moreno, 2023). Consequently, exploring metacognitive processes provides a more nuanced understanding of how engagement leads to performance success among advanced music students.

Three dimensions of metacognition—cognitive confidence, need to control thoughts, and positive beliefs about worry—were selected as mediators in this study because they represent complementary facets of metacognitive functioning. Cognitive confidence reflects the belief in one’s memory and attentional abilities, which enhances strategic planning and monitoring during practice. Need to control thoughts captures the regulatory aspect of metacognition, relating to the management of intrusive or distracting thoughts, which is especially relevant in high-pressure performance environments (Papageorgi et al., 2010). Positive beliefs about worry represent the motivational component, encompassing the perception that moderate concern can foster preparedness and sustained effort (Dweck, 2006; Osborne et al., 2020).

These three dimensions collectively correspond to the cognitive, regulatory, and motivational layers of metacognitive functioning. Other subscales of the MCQ-30, such as negative beliefs about uncontrollability or cognitive self-consciousness, were excluded because they capture maladaptive or introspective elements less pertinent to performance contexts. By integrating these dimensions into the broader theoretical frameworks of metacognition and self-regulation, the present study offers a comprehensive view of the psychological mechanisms through which musical engagement is transformed into performance achievement.

## 3 Literature review and hypotheses development

### 3.1 Engagement and performance outcomes

Building on the theoretical framework described above, engagement in music learning can be understood not only as behavioral participation but also as a motivational and cognitive state that channels effort toward mastery. Engagement, characterized by sustained attention, emotional investment, and dedication to practice, is essential for skill development and artistic expression (Allred, 2019). In music education, engagement reflects the student’s capacity to remain focused, resilient, and emotionally connected to the learning process, forming the motivational foundation of self-regulated learning (Zimmerman and Kitsantas, 1997). Previous studies have linked high levels of engagement to improved technical and interpretative skills in musicians, indicating that students who are more engaged in their practice routines tend to achieve superior performance outcomes (Lehmann and Ericsson, 1997). Studies focusing on Chinese music students have revealed that both social and educational factors shape their music engagement. For example, research in Shanghai has demonstrated that students’ musical experiences are intertwined with societal influences and their learning environments (Ho and Law, 2006). Additionally, engagement in music education that incorporates both popular and traditional elements has been shown to influence students’ preferences and enrich their learning experiences (Ho, 2017). Together, these studies indicate that engagement functions as a proactive and motivational process through which learners transform sustained practice effort into tangible performance outcomes. This evidence supports the hypothesis that music students’ engagement directly predicts music performance outcomes.

Hence, the following research hypothesis is proposed:

*RH1*: Music students’ engagement directly predicts music performance outcomes.

### 3.2 Metacognition and its mediating role

Metacognition, understood as the awareness, monitoring, and regulation of one’s own cognitive and emotional processes, plays a central role in shaping learning and performance outcomes. It has been conceptualized as a multi-faceted construct that includes both knowledge about cognition and the active regulation of cognitive strategies (Hallam, 2001; Wells and Cartwright-Hatton, 2004). Within music education, metacognitive processes enable students to plan, monitor, and evaluate their practice routines, facilitating more deliberate and efficient learning. In the Chinese context, such strategies have been shown to enhance students’ ability to self-correct and sustain motivation, ultimately improving the effectiveness of their engagement (Zimmerman and Kitsantas, 1997).

Building on the theoretical model introduced earlier, the present study proposes that metacognitions function as underlying mechanisms that explain how musical engagement translates into performance achievement. Prior evidence indicates that music education fosters both positive achievement emotions and intrinsic motivation, which are closely related to metacognitive regulation (Zachariou et al., 2023). This suggests that metacognition operates as a bridge between students’ motivation and their learning outcomes, guiding how engagement is transformed into measurable performance success.

Accordingly, the following hypothesis is proposed:

*RH2:* Metacognitions mediate the relationship between music students’ engagement and music performance outcomes.

### 3.3 Cognitive confidence

Cognitive confidence, defined as the belief in one’s cognitive abilities and reliability of memory and attention, represents a crucial dimension of metacognition that influences how musicians approach complex learning and performance tasks. Research has demonstrated that students who possess higher cognitive confidence are more likely to approach challenging pieces with a positive mindset and resilience (Burgoyne et al., 2016). In the context of music education, such confidence fosters a sense of control and self-efficacy, allowing students to sustain motivation and persist through performance difficulties. Similarly, previous studies have suggested that Chinese music students who display strong intrinsic motivation and value successful performances tend to experience greater emotional adjustment and higher career aspirations (Wang et al., 2024). These attributes are closely linked to cognitive confidence, which supports the development of adaptive practice strategies and resilience under pressure (Dweck, 2006).

Accordingly, cognitive confidence is expected to act as a mediating variable that strengthens the pathway between engagement and performance outcomes by enhancing persistence, focus, and perceived competence.

Hence, the hypothesis proposed is:

*RH2a:* Cognitive confidence mediates the relationships between music students’ engagement and music performance outcomes.

### 3.4 Need to control thoughts

The need to control thoughts refers to an individual’s perceived necessity to suppress, manage, or regulate intrusive or negative cognitions. This dimension of metacognition is particularly relevant in performance environments that demand sustained concentration and emotional stability, where intrusive thoughts can disrupt attention and impair execution (Wells and Matthews, 1994). In music performance settings, such mental intrusions often manifest as self-doubt or over-monitoring of technical details, which can interfere with flow and expression. The competitive nature of Chinese music education may amplify this tendency, requiring effective cognitive and emotional regulation strategies to transform engagement into productive and focused practice sessions (Ho and Law, 2006).

When adequately regulated, the need to control thoughts can play a constructive role by maintaining attentional focus and preventing cognitive overload (Papageorgi et al., 2010). However, an excessive desire for control may paradoxically intensify anxiety and disrupt performance, highlighting the importance of adaptive thought management rather than suppression. Within this study’s model, this dimension captures the regulatory function of metacognition, reflecting how students’ efforts to control their thoughts mediate the relationship between engagement and performance.

Based on this evidence, the following hypothesis is proposed:

*RH2b:* The need to control thoughts mediates the relationships between music students’ engagement and music performance outcomes.

### 3.5 Positive beliefs about worry

Positive beliefs about worry refer to the perception that worrying can serve functional and motivational purposes, such as preparing for potential challenges or enhancing readiness for demanding situations (Wells and Cartwright-Hatton, 2004). Within music performance, these beliefs may help students reinterpret pre-performance anxiety as a constructive signal that promotes preparation and focus. Students who hold such beliefs are often able to channel their engagement into productive forms of practice, using their concerns as motivation to refine their skills and anticipate possible difficulties during performance (Osborne et al., 2020).

In the Chinese educational context, where perseverance and diligence are highly valued, positive beliefs about worry may align with culturally endorsed attitudes toward effort and self-improvement. The integration of traditional and contemporary teaching practices (Cheung, 2004) and culturally informed pedagogy (Ho, 2017) can further nurture adaptive interpretations of worry, transforming it into a motivational resource that supports both engagement and achievement.

Accordingly, this dimension represents the motivational component of metacognition within the proposed model, illustrating how adaptive beliefs about worry can mediate the link between engagement and performance outcomes.

Hence, in the present study, the last hypothesis is

*RH2c:* The Positive beliefs about worry mediate the relationships between music students’ engagement and music performance outcomes.

Collectively, these theoretical insights and empirical findings from studies conducted in China highlight the nuanced roles that metacognitive components play in shaping the effectiveness of music students’ engagement. By analyzing how cognitive confidence, the need to control thoughts, and positive beliefs about worry function as cognitive, regulatory, and motivational mechanisms, this study aims to provide a comprehensive and theoretically grounded understanding of the psychological processes through which engagement contributes to musical achievement in Chinese higher education.

**FIGURE 1:**
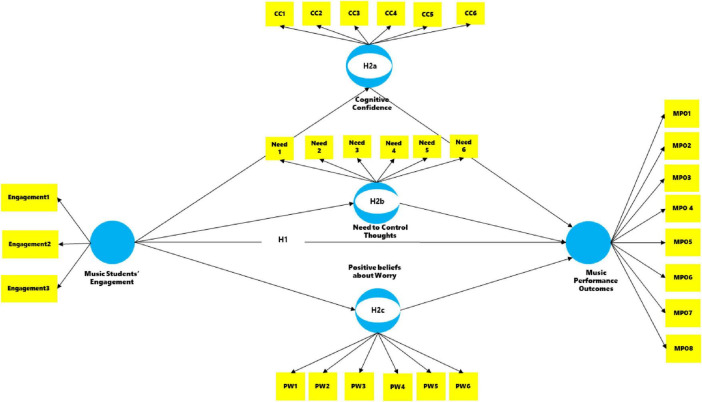
PLS structural model.

The comprehensive set of hypotheses is visually presented in Figure 1.

## 4 Research method

### 4.1 Data collection procedure and sample

As the study aimed to test the hypotheses among advanced music students, the inclusion criteria were defined as being a music student with at least 4 years of music education. Information about the research was disseminated through popular social networks, including WeChat and Weibo, to recruit participants. These platforms were selected due to their widespread use among students and young professionals, making them effective channels for reaching the target group. WeChat’s versatile functions, such as direct messaging and public group posts, allowed for targeted recruitment, while Weibo provided a platform for broadcasting participation invitations and engaging a broader audience. Data collection was carried out using Wenjuanxing (wjx. cn), a widely used web-based survey platform in China. This platform is known for its user-friendly interface and reliable functionality, which facilitated seamless data collection. Wenjuanxing supports a range of question formats and provides strong data security, ensuring that participants can easily access and complete the survey from both desktop and mobile devices. Before initiating the study, ethical approval was obtained from the Ethics Committee of the (masked for review) University, ensuring adherence to ethical research practices. Participants were provided with detailed information about the study’s purpose and methodology and gave informed consent, which included permission for data collection and the publication of results. This structured, ethically compliant approach ensured the reliability and validity of the collected data. The data are publicly available under the Creative Commons 4.0 License agreement in the OSF project of the study (reviewers’ link: https://tinyurl.com/y6x5bkv5).

The study sample consisted of 1486 advanced music students from various conservatories in China. The participants’ ages ranged from 18 to 37 years, with an average age of 23.23 (SD = 4.63). The mean number of years of previous music training was 9.29 (SD = 4.65), with a range spanning from 4 to 28 years of music education experience. The gender distribution was 59.9% male (*n* = 890) and 40.1% female (*n* = 596). Participants specialized in various areas within music, with the largest group being string instrument students (26.7%, *n* = 397), followed by those specializing in vocal performance (24.2%, *n* = 360) and piano (13.5%, *n* = 200). Other specialties included guitar (6.5%, *n* = 97), harp (8.9%, *n* = 132), brass (5.9%, *n* = 88), woodwinds (2.7%, *n* = 40), percussion (2.3%, *n* = 34), and orchestral performance (9.3%, *n* = 138). Regarding educational background, 61.5% of the participants (*n* = 914) were enrolled in college programs, while 38.5% (*n* = 572) were pursuing postgraduate studies, including master’s and doctoral programs. The majority of participants (61.6%, *n* = 916) were attending public conservatories, with the remaining 38.4% (*n* = 570) enrolled in private institutions. In terms of employment status, most participants were full-time students (87.8%, *n* = 1305). A smaller portion reported part-time work (9.8%, *n* = 146), 1.5% (*n* = 23) were employed full-time, and 0.8% (*n* = 12) were actively searching for employment.

### 4.2 Measurements

#### 4.2.1 Music students’ engagement

Music students’ engagement was measured using an ultra-short 3-item version of the Utrecht Work Engagement Scale-Student Form (UWES-SF). This condensed version includes one item representing each dimension of the original scale and has been shown to be a reliable indicator of student engagement, with a Cronbach’s α of 0.86. This scale effectively captures the overall level of engagement in student activities and has been validated in previous research. The use of the ultra-short version is supported by findings from Gusy et al. (2019), where the scale demonstrated its reliability and utility in assessing student engagement across different academic contexts. Using back-translation procedures, the three items of the scale have been translated to Chinese by the authors (Bennett, 2022).

#### 4.2.2 Metacognitions

Metacognitions were assessed using the Meta-Cognitions Questionnaire 30 (MCQ-30), developed by Wells and Cartwright-Hatton (2004), and adapted to Chinese (Zhang et al., 2020). The MCQ-30 is a 30-item instrument created by selecting six representative items for each of the five factors identified in the original Meta-Cognitions Questionnaire based on criteria such as factor loadings. This questionnaire has demonstrated a five-factor structure similar to the original scale. It has shown adequate psychometric properties across various studies using confirmatory factor analyses and large population samples, including research by several authors, both applied to clinical and general populations (Cook et al., 2014; Dethier et al., 2017; Fisher et al., 2016; Huntley et al., 2022; Martin et al., 2014). The MCQ-30 includes subscales that measure different aspects of metacognitive beliefs. For this study, the subscales used were positive beliefs about worry, which assesses the extent to which individuals believe that worry is beneficial; cognitive confidence, which evaluates confidence in one’s attention and memory processes; and beliefs about the need for control, which assesses the perceived necessity to control or suppress certain thoughts. Moreover, the questionnaire has been applied in non-clinical populations to analyze the implications of metacognitive beliefs. Recent studies have confirmed the internal consistency and structure of the MCQ-30 in Chinese versions for undergraduate University students (Zhang et al., 2020) and for adolescents (Li et al., 2025). Although originally developed for clinical applications, the MCQ-30 has been widely validated in non-clinical and educational populations, including university students and musicians (Huntley et al., 2022; Zhang et al., 2020; Araújo et al., 2024). Its subscales measure general metacognitive beliefs—such as confidence in cognitive abilities, perceived need to control thoughts, and beliefs about the usefulness of worry—that transcend specific contexts. This theoretical breadth makes the instrument appropriate for examining cognitive and emotional regulation in performance-oriented domains such as music. In the present study, only three subscales—Cognitive Confidence, Need to Control Thoughts, and Positive Beliefs about Worry—were selected because they capture the core components of self-regulation that are most relevant to musical practice and performance. This selective use of the MCQ-30 follows previous research emphasizing the adaptive and domain-general nature of metacognitive processes in learning environments, while avoiding the maladaptive dimensions that are primarily clinical in focus.

#### 4.2.3 Performance outcome (music performance quality)

The measure for performance outcomes was Music Performance Quality (MPQ), assessed as a self-reported measure using the MPQ Scale (Nielsen et al., 2018). The MPQ scale comprises nine dimensions, which include “tempo,” “rhythm,” “intonation,” “tone,” “dynamics,” “articulation,” “musical understanding and interpretation,” “missing notes, wrong notes and unwritten breaks,” and “global appreciation.” Each dimension is rated on a 5-point scale ranging from 1 (lowest score) to 5 (highest score). Each dimension includes a clear definition and specific criteria for scoring, ensuring consistent evaluation. The final MPQ score is computed as the average of all nine dimension scores, with higher scores indicating better music performance quality.

### 4.3 Data analytic strategy

Descriptive and correlation analyses were performed using SPSS version 29.0, while structural equation modeling (SEM) was conducted with SmartPLS 4 (Ringle et al., 2024). Initially, descriptive statistics were calculated in SPSS 29.0 to provide an overview of the sample characteristics. This included computing means, standard deviations, and frequency distributions for demographic variables such as age, gender, education level, music specialty, and employment status. These analyses offered insights into the participants’ overall profile and helped establish a context for further statistical exploration. Pearson’s correlation analyses were also performed in SPSS 29.0 to assess the relationships between the main study variables, including student engagement (measured by the ultra-short version of the Utrecht Work Engagement Scale—Student Form), metacognitive beliefs (measured using subscales from the Meta-Cognitions Questionnaire 30), and music performance outcomes (measured by the Music Performance Quality scale). The significance of correlations was evaluated to identify potential associations and inform subsequent structural modeling. Structural equation modeling (SEM) was conducted using SmartPLS 4 to test the hypothesized direct and mediated relationships between constructs. Path coefficients were examined to assess the strength and significance of these relationships, while indirect effects were evaluated to determine the mediating roles of metacognitive beliefs—specifically cognitive confidence, positive beliefs about worry, and the need for control—between student engagement and performance outcomes. Model fit was evaluated using the Standardized Root Mean Square Residual (SRMR) and the Normed Fit Index (NFI). Collinearity among variables was checked using the Variance Inflation Factor (VIF) to ensure that multicollinearity did not affect the stability of the results. The R-square and adjusted R-square values were reported to indicate the variance explained by the predictors for each dependent variable, and f-square values were calculated to assess the effect sizes within the model. Bootstrapping techniques were employed in SmartPLS 4 to test the significance of path coefficients and mediation effects, ensuring robustness and reliability in the results. All statistical tests were conducted with a significance threshold set at *p* < 0.05, with notable findings highlighted at both the 0.01 and 0.05 levels. This comprehensive analytical strategy provided a detailed understanding of the relationships between engagement, metacognitive beliefs, and performance outcomes among advanced music students.

## 5 Results

### 5.1 Descriptive and correlational analyses

The descriptive statistics for the main constructs in the study, including means, standard deviations, and sample sizes, are presented in Table 1. The analysis was based on data from 1486 participants. The mean values ranged from 2.42 (Need Control and Positive Beliefs) to 2.97 (Cognitive Confidence), with standard deviations between 0.63 (Cognitive Confidence) and 0.98 (Music Students’ Engagement), indicating a moderate level of variability in the responses. Table 1 also presents the Pearson correlation coefficients among the constructs. The correlations indicate significant relationships between various constructs at different levels of strength. Notably, Music Students’ Engagement and Performance Outcomes showed a moderate positive correlation (*r* = 0.300, *p* < 0.001), suggesting that higher engagement is associated with better performance outcomes. Cognitive Confidence was positively correlated with Performance Outcomes (*r* = 0.206, *p* < 0.001) and strongly associated with Positive Beliefs (*r* = 0.448, *p* < 0.001). Need Control displayed a weak correlation with other variables, except for a notable positive correlation with Positive Beliefs (*r* = 0.388, *p* < 0.001).

**TABLE 1:** Descriptive statistics and correlations below the median.

Construct	Mean	SD	1.	2.	3.	4.	5.
1. Music students’ engagement	2.73	0.98	–	–	–	–	–
2. Cognitive confidence	2.96	0.63	0.224**				
3. Need control	2.42	0.90	−0.004	0.077**			
4. Positive beliefs	2.42	0.73	0.093**	0.448**	0.388**		
5. Performance outcomes	2.83	0.75	0.300**	0.206**	0.020	0.065*	

*N* = 1486. Correlations marked with ** are significant at the 0.01 level (two-tailed). Correlations marked with * are significant at the 0.05 level (two-tailed).

### 5.2 Common method bias

To ensure that the results of this study were not significantly influenced by common method bias, a collinearity assessment was conducted. This step is crucial for verifying that the measurement model’s items do not exhibit high multicollinearity, which could skew the interpretation of relationships within the model. The collinearity statistics were assessed by calculating Variance Inflation Factor (VIF) values for both the outer and inner model constructs. VIF values are essential for detecting multicollinearity, and a general rule is that VIF values below 5 indicate an acceptable level of collinearity among items. In the outer model, all items demonstrated acceptable VIF values, ranging from 1.120 to 3.924. This range indicates that collinearity among the measured indicators does not pose a significant threat to the analysis.

The inner model collinearity assessment showed that all VIF values fell within acceptable limits. The highest VIF observed was 1.335 for the relationship between Positive Beliefs about Worry and Performance Outcomes, while the lowest was 1.000 for the paths from Music Students’ Engagement to the other constructs. The consistency of these results confirms that there is no problematic collinearity that could compromise the validity of the model’s structural paths. In summary, the collinearity diagnostics indicate that multicollinearity is not an issue for either the outer or inner models. This supports the robustness of the measurement model, ensuring reliable interpretations in subsequent sections of this study. Table 2 presents statistical details for VIF values.

**TABLE 2:** Variance inflation factor (VIF) values for outer and inner models.

Item/path	VIF
**Outer model**	
Engagement 1	1.405
Engagement 2	1.803
Engagement 3	1.776
CC1	1.543
CC2	2.166
CC3	2.290
CC4	2.163
CC5	1.360
CC6	1.495
Need1	2.884
Need2	3.924
Need3	3.484
Need4	2.360
Need5	2.956
Need6	1.709
PW1	1.304
PW2	2.003
PW3	2.007
PW4	1.214
PW5	1.730
PW6	1.777
MPO1	2.191
MPO2	2.616
MPO3	1.434
MPO4	1.692
MPO5	1.953
MPO6	2.145
MPO7	1.752
MPO8	1.610
**Inner model**	
Cognitive confidence → performance outcomes	1,213
Need control thoughts → performance outcomes	1,132
Positive beliefs about worry → performance outcomes	1,284
Music students engagement→ cognitive confidence	1,000
Music students engagement→ need control thoughts	1,000
Music students engagement→ performance outcomes	1,080
Music students engagement→ positive beliefs about worry	1,000

VIF values below 5 indicate acceptable levels of multicollinearity.

### 5.3 Model fitness

The model fit assessment provides an understanding of how well the proposed model represents the observed data. Several fit indices were analyzed to evaluate the overall fit of both the saturated and estimated models. The standardized root mean square residual (SRMR) value for the saturated model was 0.087, while for the estimated model, it was 0.114. These values, though higher than the ideal threshold of 0.08 for a good fit, still provide insight into the closeness of the data to the model predictions. For the discrepancy measures, the d_ULS value was 3.328 for the saturated model and increased to 5.636 in the estimated model. Similarly, the d_G value moved from 0.904 in the saturated model to 0.956 in the estimated model. These metrics reflect the differences in discrepancies between the covariance matrices. The Chi-square statistic for the saturated model was 7421.027 and increased to 7774.662 in the estimated model. While higher Chi-square values generally indicate a poorer fit, these results should be considered alongside other fit indices due to the sensitivity of Chi-square to sample size. The Normed Fit Index (NFI) was 0.658 for the saturated model and 0.642 for the estimated model. Although an NFI value closer to 1 suggests a better fit, these values indicate that the model’s fit could be improved. Overall, the model fit indices suggest that while the model provides an acceptable representation of the data, some refinement may be needed to enhance the fit. Full statistical details are provided in the fit summary table.

### 5.4 Construct reliability and convergent validity

Assessing construct reliability and convergent validity is crucial to ensuring that the constructs are reliable and valid. This analysis was conducted using Cronbach’s alpha, composite reliability (rho_a and rho_c), and the average variance extracted (AVE). The construct reliability for each latent variable was evaluated using Cronbach’s alpha and composite reliability. All constructs showed adequate levels of reliability (Table 3). Cognitive Confidence had a Cronbach’s alpha of 0.804, composite reliability (rho_c) of 0.857, and an AVE of 0.506. Need control Thoughts displayed strong reliability, with a Cronbach’s alpha of 0.917, composite reliability of 0.918, and an AVE of 0.656, meeting the threshold for convergent validity. Performance Outcomes reported a Cronbach’s alpha of 0.805, composite reliability of 0.850, but an AVE of 0.420, indicating acceptable reliability but potential issues with convergent validity. Positive beliefs about worry had a Cronbach’s alpha of 0.774, composite reliability of 0.838, and an AVE of 0.472, suggesting marginal convergent validity. Music Students Engagement demonstrated strong construct reliability with a Cronbach’s alpha of 0.775, composite reliability of 0.864, and an AVE of 0.680. The AVE values were examined for convergent validity. For a construct to exhibit adequate convergent validity, the AVE should be greater than 0.50. While Need to Control Thoughts and Music Students’ Engagement met this criterion, other constructs, such as Cognitive Confidence, Performance Outcomes, and Positive Beliefs about Worry, had AVE values below 0.50, indicating that these constructs might require further refinement.

**TABLE 3:** Construct reliability and convergent validity.

Construct	Cronbach’s alpha	Composite reliability (rho_a)	Composite reliability (rho_c)	Average variance extracted (AVE)
Cognitive confidence	0.804	0.838	0.857	0.506
Need control thoughts	0.917	0.689	0.919	0.658
Performance outcomes	0.805	0.826	0.850	0.420
Positive beliefs about worry	0.774	0.868	0.838	0.472
Music students’ engagement	0.775	0.825	0.864	0.680

### 5.5 Discriminant validity

Discriminant validity was assessed using the Heterotrait-Monotrait ratio (HTMT), Fornell-Larcker criterion, and cross-loadings. The HTMT values for all construct pairs were below the recommended threshold of 0.90, supporting the discriminant validity between constructs (Henseler et al., 2015). The highest HTMT value was 0.566 between Cognitive Confidence and Positive beliefs about worry, which still fell within acceptable limits (Table 4). The Fornell-Larcker criterion results indicated that the square root of the AVE for each construct was higher than its correlation with any other construct, suggesting acceptable discriminant validity. For example, Cognitive Confidence had a value of 0.712 on the diagonal, higher than its correlations with other constructs.

**TABLE 4:** Heterotrait-Monotrait ratio (HTMT).

Construct pair	HTMT Value
Need control thoughts < - > cognitive confidence	0.124
Performance outcomes < - > cognitive confidence	0.271
Performance outcomes < - > need control thoughts	0.097
Positive beliefs about worry < - > cognitive confidence	0.408
Positive beliefs about worry < - > need control thoughts	0.457
Positive beliefs about worry < - > performance outcomes	0.196
Music students’ engagement < - > cognitive confidence	0.299
Music students’ engagement < - > need control thoughts	0.124
Music students’ engagement < - > performance Outcomes	0.385
Music students’ engagement < - > positive beliefs about worry	0.206

The analysis of cross-loadings confirmed that each indicator loaded higher on its respective construct than on any other constructs. For instance, it54 had a loading of 0.727 on Cognitive Confidence, which was higher than its loadings on other constructs.

### 5.6 Structural model

The structural model was assessed using R-square and f-square values to evaluate the predictive power and effect sizes of the constructs in the model (Tables 5, 6).

**TABLE 5:** R-squared and R-squared adjusted values.

Construct	R-square	R-square adjusted
Cognitive confidence	0.070	0.069
Need control thoughts	0.004	0.004
Performance outcomes	0.143	0.140
Positive beliefs about worry	0.019	0.018

**TABLE 6:** f-squared values.

Path	f-square
Cognitive confidence - > performance outcomes	0.033
Need control thoughts - > performance outcomes	0.002
Positive beliefs about worry - > performance outcomes	0.0
Music students’ engagement- > cognitive confidence	0.074
Music students’ engagement- > need control thoughts	0.004
Music students’ engagement- > performance outcomes	0.082
Music Students’ Engagement- > Positive beliefs about worry	0.019

### 5.7 R-Square overview

The R-square values indicate the proportion of variance explained by the model for each dependent construct. The adjusted R-square accounts for the number of predictors in the model, providing a more balanced measure, as table shows.

These results suggest that performance outcomes have the highest proportion of variance explained by the predictors, followed by cognitive confidence. Need control thoughts and positive beliefs about worry show low explained variance, indicating limited predictive power from the current model.

### 5.8 F-square values

The f-square values were analyzed to assess the effect size of each path within the structural model. These values help determine the impact of one construct on another:

Effect sizes are categorized as small (0.02), medium (0.15), and large (0.35). The f-square results indicate that Music Students’ Engagement has a moderate effect on performance outcomes and cognitive confidence, while most other paths have small or negligible effects.

These results provide insights into which constructs are the main contributors to the explained variance and the strength of the relationships in the model. The details are summarized in the provided tables for further reference.

### 5.9 Hypothesis testing

#### 5.9.1 Direct relationships

The direct relationships between constructs were evaluated using path coefficients. The path coefficients are presented as follows in Table 7.

**TABLE 7:** Direct, indirect, and total path coefficients.

Path	Path coefficient
Cognitive confidence - > performance outcomes	0.192
Need control thoughts - > Performance outcomes	0.047
Positive beliefs about worry - > performance outcomes	0.002
Music Students’ Engagement - > cognitive confidence	0.263
Music students’ engagement - > need control thoughts	0.065
Music students’ engagement - > performance outcomes	0.275
Music students’ engagement - > Positive beliefs about worry	0.137
**Mediated path**	**Specific** **indirect** **effect**
Music students’ engagement - > positive beliefs about worry - > performance outcomes	0.000
Music students’ engagement - > need control thoughts - > performance outcomes	0.003
Music students’ engagement - > cognitive confidence - > performance outcomes	0.049
**Path**	**Total** **effect**
Cognitive confidence - > performance outcomes	0.192
Need control thoughts - > performance outcomes	0.046
Positive beliefs about worry - > performance outcomes	0.002
Music students’ engagement - > cognitive confidence	0.265
Music students’ engagement - > need control thoughts	0.065
Music students’ engagement - > performance outcomes	0.327
Music students’ engagement - > Positive beliefs about worry	0.135

The results indicate that Music Students’ Engagement has a strong direct positive effect on Performance Outcomes (path coefficient = 0.275), supporting hypothesis H1. The relationships between Cognitive Confidence (0.192) and the Need to control Thoughts (0.046) with Performance Outcomes suggest weaker but positive effects, while Positive beliefs about worry show a negligible effect (0.002).

#### 5.9.2 Mediated relationships

The mediation effects were tested to explore how intermediate constructs influence the relationship between Music Students’ Engagement and Performance Outcomes through metacognitive constructs. H2a is supported by the indirect effect of Music Students’ Engagement on Performance Outcomes through Cognitive Confidence, which is positive (0.050). H2b has a minor mediated effect (0.003) through the Need to Control Thoughts, and H2c shows a negligible effect (0.002) through Positive beliefs about worry.

**FIGURE 2:**
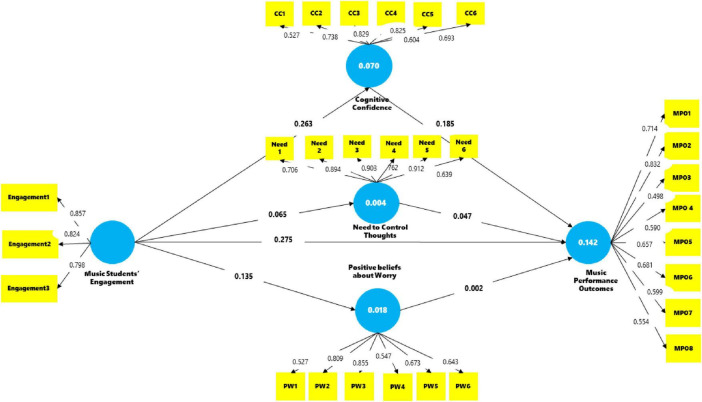
The structural model with path coefficients.

The total effects include both direct and indirect effects, summarizing the overall impact of Music Students’ Engagement on the constructs, which are displayed in Table 7. Figure 2 shows the path coefficients for the PLS analyses.

## 6 Discussion

The present study aimed to test the relationships between music students’ engagement, metacognitive factors, and performance outcomes among Chinese advanced music students. While the findings generally support the hypotheses, certain results warrant a closer discussion to fully understand their implications. In addition to confirming the predictive role of engagement, this study proposes a theoretically grounded framework in which metacognitive processes—cognitive confidence, thought regulation, and adaptive worry—serve as key mechanisms linking motivation to performance. This integration of metacognitive and self-regulated learning theories extends previous research by offering a unified explanation of how engagement translates into achievement within the context of music education.

The results of this study provide robust support for H1: Music Students’ Engagement directly predicts Music Performance Outcomes. The positive and significant path coefficient underscores the strong effect of engagement on performance outcomes among Chinese advanced music students. This finding is consistent with the broader literature that emphasizes the integral role of engagement in fostering proactive learning and enhancing performance (Chen, 2024). Engagement allows students to immerse themselves in their practice, driven by intrinsic motivation and alignment with their values (Tu and Fu, 2024). This proactive approach helps students build the resilience and skills necessary for effective performance. Engagement is not merely about time spent practicing; it encompasses a state of vigor, dedication, and absorption (Wu and Qin, 2025). These attributes collectively contribute to realizing a student’s potential and enhancing their coping mechanisms for the demands of music-making. The study’s confirmation of H1 aligns with Concina (2019), which highlights that students with high metacognitive abilities linked to their engagement levels can better structure their practice and learning strategies, ultimately achieving more successful outcomes. Addressing these metacognitive dimensions in music education can further strengthen students’ strategic behaviors and optimize their learning processes. However, the study also reveals interesting nuances regarding the influence of specific metacognitive factors. Cognitive confidence demonstrates a positive but less pronounced relationship with performance outcomes. This indicates that belief in one’s cognitive abilities supports effective practice and performance. But, it does not carry as substantial an impact as general engagement. The findings align with prior research that engaged students tend to experience “flow” states, where they are deeply absorbed in their activities (O’Neill, 1999). These experiences are essential for maintaining long-term interest and progress in music, which moderate achievers often miss out on, hindering their development and performance outcomes (Statham, 2016).

Although the need to control thoughts is positively correlated with performance, it shows a weaker effect. This suggests that while managing intrusive thoughts can contribute to performance, excessive focus on control may potentially impede more fluid and adaptive practice methods. Such findings are in line with studies indicating that competitive or overly pressured environments can reduce engagement and negatively impact outcomes, even when students are otherwise motivated (O’Neill, 1999). A supportive and encouraging peer environment, on the other hand, fosters better engagement and leads to improved outcomes.

Interestingly, the path coefficient associated with positive beliefs about worry indicates an almost negligible effect. This result may seem counterintuitive at first but can be explained by the nature of worry as a double-edged sword. While moderate worry can motivate preparation and improvement, excessive or misplaced positive beliefs about worry could contribute to maladaptive practices or unnecessary stress, detracting from optimal engagement and performance (Concina, 2019).

These differentiated patterns among the three metacognitive variables are consistent with the theoretical distinction between adaptive and potentially maladaptive regulation processes proposed by Wells and Matthews (1994). They suggest that only those metacognitive dimensions that directly facilitate self-regulated learning—such as cognitive confidence—meaningfully contribute to performance, whereas overly controlling or worry-based strategies may interfere with engagement’s positive effects.

Overall, this study reinforces the critical role of engagement in enhancing performance outcomes while showing that metacognitive competencies like cognitive confidence and the need to control thoughts play secondary, supportive roles. Addressing these factors in educational practices by creating supportive environments and promoting strategic, metacognitive-focused learning can help students reach their full potential in music performance. The results emphasize that engagement must be viewed as a dynamic state influenced by both personal and contextual factors, aligning with the conclusions of Tu and Fu (2024) and others who advocate for balancing engagement with well-being for sustained performance success.

The mediation analyses confirmed H2a, showing that cognitive confidence mediates the relationship between music students’ engagement and performance outcomes with a positive indirect effect. This supports prior research emphasizing the role of metacognition in planning, regulating, and assessing cognitive processes, which are crucial for effective learning and performance (Palmer and Drake, 1997). High cognitive confidence allows students to engage in self-regulation—planning, monitoring, and adjusting their practice strategies, leading to more structured and successful outcomes (Araújo et al., 2024). This aligns with the notion that metacognitive awareness fosters strategic behavior in musicians, enabling them to fulfill performance tasks efficiently (Bonnaire and González-Moreno, 2023).

H2b, concerning the need to control thoughts, showed a minor mediated effect. This finding, while positive, indicates that this metacognitive component plays a limited role in influencing performance outcomes. The literature suggests that metacognitive skills facilitate self-monitoring and control, allowing musicians to manage intrusive thoughts and maintain focus (Araújo et al., 2024). However, excessive effort to control thoughts can sometimes lead to cognitive overload, which might explain the weaker effect observed. Supportive environments that reduce the pressure to control thoughts and encourage reflective learning can help optimize this balance (Fazil et al., 2024).

H2c showed a negligible effect for the mediation of positive beliefs about worry. This suggests that while a degree of worry can motivate preparation, an overreliance on positive beliefs about worry may not translate into improved performance. Research indicates that while reflective thinking and a certain level of concern can guide musicians to analyze their strengths and weaknesses and adjust accordingly, excessive worry may disrupt self-regulatory processes and hinder performance (Araújo et al., 2024). This outcome is supported by studies on cue utilization and self-efficacy, which highlight that metacognitive judgments and self-beliefs must be balanced to effectively guide practice (Peynircioğlu et al., 2014).

Together, these findings partially confirm the metacognitive model proposed in this study, suggesting that only adaptive forms of regulation—particularly cognitive confidence—function as genuine mediators between engagement and performance. Overall, the total effects of engagement on performance outcomes, considering both direct and indirect paths, underscore the multifaceted role of engagement in music practice. While engagement itself drives proactive learning and supports performance (Upitis et al., 2017), metacognitive constructs contribute nuanced, context-dependent impacts. High cognitive confidence emerges as a vital component that enhances strategic planning and monitoring, directly benefiting performance (Araújo et al., 2024; Hallam, 2001). On the other hand, the need to control thoughts and positive beliefs about worry, while beneficial in moderation, must be managed carefully to prevent potential negative effects on learning processes and performance.

These results thus support the theoretical integration of metacognition and self-regulated learning frameworks (Wells and Matthews, 1994; Zimmerman and Kitsantas, 1997), demonstrating that engagement acts as a motivational driver that operates through specific cognitive mechanisms to produce measurable performance gains.

This study’s findings reinforce the importance of integrating metacognitive training in music education. Educators should focus on fostering cognitive confidence and balanced strategies that incorporate self-reflection and adaptive responses. Emphasizing planning and strategy use, alongside regular monitoring and evaluation of practice sessions, can lead to better performance outcomes (Concina, 2019). By supporting students’ metacognitive development, music programs can help them navigate the complexities of practice and performance, fostering resilience, strategic behavior, and academic success, as well as in other educational areas in which performance outcomes are specifically relevant (Love et al., 2019a).

### 6.1 Limitations of the present study and suggestions for future research

While the present study provides valuable insights into the relationships between music students’ engagement, metacognitive beliefs, and performance outcomes, several limitations should be acknowledged. One major limitation is the cross-sectional design, which limits the ability to draw causal inferences. Although structural equation modeling was employed to test the relationships among variables, the data were collected at a single point in time, making it difficult to establish definitive cause-and-effect relationships. Future research could benefit from a longitudinal design that tracks changes in engagement, metacognitive beliefs, and performance outcomes over time to better understand causality and developmental patterns in these constructs.

Another area for improvement lies in the use of self-reported measures, which may introduce bias due to social desirability or inaccuracies in self-assessment. While the instruments used, such as the Chinese version of the Meta-Cognitions Questionnaire 30 (MCQ-30) (Li et al., 2025) and the ultra-short version of the Utrecht Work Engagement Scale—Student Form (Gusy et al., 2019), have been validated in prior studies and demonstrated good psychometric properties, the reliance on self-reported data may not capture the full scope of participants’ engagement or metacognitive processes. Future research could incorporate objective performance assessments or external evaluations to complement self-reported data and enhance the robustness of the findings (Nielsen et al., 2018). The sample for this study was limited to advanced music students in China, which may affect the generalizability of the results. While the sample size was substantial and included a diverse range of music specialties, the cultural and educational context of Chinese conservatories may differ from that of other countries (Spada et al., 2008). Future studies should consider including samples from different cultural and educational backgrounds to determine whether the observed relationships hold across various contexts.

Additionally, the study focused on a limited range of metacognitive beliefs, specifically cognitive confidence, positive beliefs about worry, and the need for control. Although these factors provide meaningful insights, metacognition is a broad construct with additional dimensions that could be relevant in the context of music performance. Future research could explore other aspects of metacognition, such as negative beliefs about worry and cognitive self-consciousness, to obtain a more comprehensive understanding of how these factors impact performance, as well as the influence of other variables, such as age-related changes (Reifinger, 2016). Moreover, while the study included various specialties within instrumental and vocal performance, further differentiation within these groups (e.g., comparisons between soloists and ensemble performers) might reveal additional nuances in how engagement and metacognitive beliefs affect performance outcomes. Investigating these sub-groups could offer a deeper understanding of the specific challenges and cognitive strategies employed by different types of music students.

Another limitation concerns the use of the MCQ-30, a measure originally developed for clinical contexts. Although its psychometric validity in non-clinical and educational samples has been supported by recent studies (Huntley et al., 2022; Zhang et al., 2020), it was not specifically designed for music learning. In this study, three theoretically relevant subscales—Cognitive Confidence, Need to Control Thoughts, and Positive Beliefs about Worry—were used as an initial, conceptually guided approach to adaptive metacognition in musical settings. Future work should incorporate domain-specific instruments that capture the distinctive metacognitive demands of music training (e.g., Concina, 2019; Bonnaire and González-Moreno, 2023) and consider mixed-method designs (e.g., interviews, reflective journals, observational data) to examine students’ metacognitive experiences in greater depth and cultural context. These designs would allow the exploration of musicians’ subjective experiences and contextual influences, offering richer insights into the mechanisms that shape engagement and performance.

Future research should therefore prioritize longitudinal designs to clarify the temporal ordering of engagement, metacognition, and performance; include objective performance indicators (e.g., juried assessments, teacher ratings) alongside self-reports; broaden sampling across cultural and institutional contexts to test the robustness of effects; examine additional metacognitive dimensions (e.g., negative beliefs about worry, cognitive self-consciousness) and moderators such as age and specialization; and analyze sub-groups (e.g., soloists vs. ensemble performers) to identify profile-specific challenges and strategies.

### 6.2 Theoretical implications

The construct of metacognitive knowledge encompasses an understanding of one’s cognitive processes, including declarative (knowledge about facts), procedural (knowledge about how to do things), and conditional (knowledge about when and why to apply specific strategies) knowledge (de Palo et al., 2017). These aspects of metacognition are vital for musicians as they navigate their practice and performance, tailoring their learning strategies to optimize outcomes. Research has consistently shown that skills such as planning, monitoring, and evaluating are essential for self-regulated learning in music (Burin and Osorio, 2016). By incorporating these skills into their practice routines, musicians can effectively manage their learning processes and adjust their methods based on progress and challenges. Later theoretical advances also incorporate the concept of embodied cognition, which posits that physical interactions with musical instruments are interwoven with cognitive processes (Hallam, 2001). This perspective highlights that musicians’ bodily experiences, such as hand movements and posture, influence their cognitive strategies, offering a more integrated approach to understanding how metacognition operates in music education. Such insights suggest that music educators should focus on both mental and physical aspects of musical practice to foster deeper metacognitive awareness (Love et al., 2019b).

### 6.3 Suggestions for music educators

The practical recommendations derived from this study are grounded in an integrated metacognitive model that encompasses cognitive, regulatory, and motivational dimensions. Recognizing how these processes operate can help educators design teaching strategies that promote both technical mastery and reflective self-regulation among students.

Music educators can enhance their teaching practices by incorporating metacognitive strategies that foster students’ cognitive development and performance (Ojala and Pohjannoro, 2024). One effective approach is to integrate metacognitive training into the curriculum, guiding students to plan, monitor, and evaluate their practice sessions. This practice strengthens students’ self-regulation skills, enabling them to adapt their strategies as needed and improve their overall learning and performance. Promoting reflective learning is also crucial, as it encourages students to think about their learning processes, successes, and areas needing improvement (Sacchetti and Salustri, 2023). This type of reflective practice helps students gain insight into their cognitive and emotional states, supporting more adaptive and effective learning outcomes.

Educators should also emphasize strategic behavior by teaching students how to apply metacognitive knowledge during practice and performance (Havnen et al., 2024). This high-order activity ensures that students can plan, monitor, and adjust their behaviors to fulfill performance tasks successfully. Enhancing self-monitoring skills by developing students’ awareness of their cognition and self-regulation can be valuable. It is important to include exercises that build self-efficacy and motivation, as these socio-psychological factors impact students’ ability to manage their learning processes effectively (Jiang and Tong, 2024). Additionally, facilitating memory processes through metacognitive strategies such as metamemory can improve memorization, retrieval, and recall, which are vital for musical performance. Educators can implement exercises that help students understand these memory strategies, contributing to enhanced learning outcomes (Papageorgi and Economidou Stavrou, 2023). Finally, incorporating principles of embodied cognition into teaching practices can provide students with a comprehensive learning experience (Viator et al., 2024). Recognizing that physical interactions with instruments influence cognitive processes allows educators to create activities that integrate action and reflection, managing cognitive activities holistically. By implementing these approaches, music educators can foster a learning environment that supports strategic, self-regulated, and reflective learning, ultimately improving academic and performance outcomes.

## 7 Conclusion

The present study explored the relationships between music students’ engagement, metacognitive factors, and performance outcomes among advanced Chinese music students. The findings provide substantial support for the hypothesis that engagement directly predicts performance outcomes, highlighting the vital role that active participation and intrinsic motivation play in enhancing musical achievements. The study also confirmed the mediating effects of cognitive confidence and, to a lesser extent, the need to control thoughts, underscoring the nuanced role of metacognitive constructs in shaping learning and performance. While positive beliefs about worry showed a negligible effect, the results suggest that balanced metacognitive strategies are essential for fostering effective practice and performance.

These insights underscore the importance of integrating metacognitive training into music education, emphasizing strategic planning, monitoring, and self-reflection. By supporting the development of these skills, educators can enhance students’ ability to self-regulate and adapt their learning approaches, leading to more successful performance outcomes. The study’s implications suggest that fostering cognitive awareness and providing a supportive learning environment can maximize the potential of music students, equipping them with the tools needed for sustained growth and achievement.

The findings therefore extend current theory by integrating metacognitive and self-regulatory perspectives within music education, establishing a coherent framework that connects motivation, cognition, and performance as interdependent components of artistic achievement.

## Data Availability

The datasets presented in this study can be found in online repositories. The names of the repository/repositories and accession number(s) can be found below: The data supporting this study’s findings are openly available and are shared by the author as a supplementary file to this submission (view-only link for masked review: https://tinyurl.com/y6x5bkv5). The project will be public after publication acceptance.
